# Retraction: Protein Tyrosine Phosphatase 1B and Insulin Resistance: Role of Endoplasmic Reticulum Stress/Reactive Oxygen Species/Nuclear Factor Kappa B Axis

**DOI:** 10.1371/journal.pone.0272823

**Published:** 2022-08-04

**Authors:** The PLOS ONE Editors

Following the publication of this article [1], the University of Wyoming informed PLOS that during institutional examination of the article, evidence was found of data irregularities and image reuse affecting Figs 5, 6, and 7, that significantly affect the results and conclusions reported in the manuscript. Specifically,

- The Fig 6A Control and TM+TUDCA panels (DAPI, DHE, and Merge) partially overlap.
- Areas indicating splicing and inappropriate image manipulation were detected in the following panels:
  ○ Fig 5A GRP78 and NCK1 panels.
  ○ Fig 7A PTP1B and GRP78 panels.

In addition to these concerns, editorial reassessment of the article raised further concerns with results presented in Figs 5, 6, 8, and 9. Specifically,

- Additional irregularities were detected in the background of the Fig 5A PTP1B panel.
- Additional similarities were detected between and within panels:
  ○ The Fig 6A TM+NAC panels (DAPI, DHE, and Merge) also appear to partially overlap with the respective Fig 6A Control and TM+TUDCA panels (DAPI, DHE, and Merge).
  ○ Fig 8A, lanes 1–4 of the Cyt. NFκB p65 panel appear similar to lanes 1–4 of the Cyt. GAPDH panel.
  ○ Fig 9C, lane 4 and lane 9 of the Cyt. Lamin A panel appear similar.

The underlying data for this article have not been submitted to the journal for editorial review.

In light of the concerns affecting multiple figure panels that question the integrity of these data, the *PLOS ONE* Editors retract this article.

SN agreed with the retraction and apologizes for the issues with the article. EP and JR either did not respond directly or could not be reached.

## References

[1] Panzhinskiy E, Ren J, Nair S (2013) Protein Tyrosine Phosphatase 1B and Insulin Resistance: Role of Endoplasmic Reticulum Stress/Reactive Oxygen Species/Nuclear Factor Kappa B Axis. PLoS ONE 8(10): e77228. 10.1371/journal.pone.0077228 24204775 PMC3799617

